# Association between suicide risk severity and sarcopenia in non-elderly Chinese inpatients with major depressive disorder

**DOI:** 10.1186/s12888-020-02763-1

**Published:** 2020-07-02

**Authors:** Xin-Xin Fan, Jing Yuan, Yu-Jun Wei, Fang Zhou, Li Xu, Yan Zhang, Jun-Yu Meng, Xiao-Long Jin, Jian-Zhong Yang

**Affiliations:** 1 Department of Psychiatry, The Second Affiliated Hospital of Kunming Medical University, 374 Dianmian Av, Kunming, 650101 Yunnan China; 2 Department of Geriatrics, The Sixth Affiliated Hospital of Kunming Medical University (People’s Hospital of Yuxi), Yuxi, Yunnan China

**Keywords:** Major depressive disorder, Sarcopenia, Suicide risk

## Abstract

**Background:**

Sarcopenia is a skeletal muscle disorder. Recent studies have shown an association between muscle health and suicide. However, there have been no previous studies on the relationship between suicide risk severity and sarcopenia in major depressive disorder (MDD). This study aimed to explore the association between suicide risk severity and sarcopenia in non-elderly Chinese inpatients with MDD.

**Methods:**

The first-episode drug-naïve MDD inpatients aged 20–59 years with the 24-item Hamilton Rating Scale for Depression (HAMD-24) scores of >20 were included, who were then classified into low, intermediate, high and very high suicide risk groups according to the Nurses’ Global Assessment of Suicide Risk (NGASR). The HAMD-24, the Hamilton Rating Scale for Anxiety (HAMA) and the SARC-F questionnaire were used to assess depression severity, anxiety severity and sarcopenia, respectively. The plasma levels of cortisol and adrenocorticotropic hormone (ACTH) were measured.

**Results:**

A total of 192 MDD inpatients (122 females, 70 males; aged 39.3 ± 11.7 years) were included, with 12.5% meeting criteria for sarcopenia. There were significant differences in gender, HAMD score and prevalence of sarcopenia among the suicide risk groups. Adjusted ordinal regression analysis showed that sarcopenia was significantly associated with more severe suicide risk (OR = 2.39, 95%CI 1.02–5.58, *p* = 0.044) independent of depression severity.

**Conclusions:**

This study revealed that sarcopenia was significantly associated with higher suicide risk in non-elderly Chinese MDD inpatients after adjustment for depression severity. Intervention of sarcopenia might be effective in reducing the risk of suicide in non-elderly MDD patients.

## Background

Suicide is the major cause of mortality worldwide. WHO reported that over 800,000 people died from suicide each year [1]. In 2018, death registration data for the UK showed that age-specific suicide rates peaked among non-elderly adults aged 20–59 years [2]. The mortality data of the US showed that suicide rates kept increasing from 1999 through 2017, especially among those aged 15–64 years [3]. The situation is even worse in patients with major depressive disorder (MDD) who present high risk of suicide and not surprisingly become the major victim of suicidality. A study showed that MDD was associated with a 20-fold increased risk of suicide [4]. There is evidence that 15% of those with MDD in community reported at least one suicide attempt in their lifetime [5], and the lifetime suicide prevalence in MDD patients was about 2–12% [6, 7]. Given the serious nature of this public health problem, researches on suicide related factors are warranted, especially among non-elderly adults with MDD.

Recently, muscle health has received increasing attention but still far less than psychosocial factors in mental health. Studies have found the association between muscle health and mental health. There is evidence suggesting associations of low muscular strength with risk of self-harm and suicide in both adolescents [8] and the elderly [9] and with death from suicide in middle-aged men [10]. Sarcopenia, a skeletal muscle disorder, is characterized with generalized decreased muscle mass and/or strength or impaired muscle function [11]. Primary sarcopenia is largely attributable to aging, while secondary sarcopenia can occur earlier in life in association with a range of other conditions [12, 13]. Recent evidence showed that sarcopenia was positively associated with depressive symptoms assessed by self-rating scales among different populations, such as community-dwelling people and patients with type II diabetes, hemodialysis, end-stage kidney disease and cancer [14–17]. Sarcopenia, suicide and depression share common risk factors, such as chronic low-grade inflammation, brain-derived neurotropic factor (BDNF) dysfunction and dysregulated hypothalamic-pituitary-adrenal (HPA) axis [18–21]. These studies suggest that the three - muscle health, depression and suicide - may connect with and overlap each other.

However, there have been few studies of sarcopenia among patients with MDD and no previous studies on the relationship between severity of suicide risk and sarcopenia in MDD.

Early identification of potential risk factors for suicide is crucial for suicide prevention in non-elderly patients with MDD. We hypothesized that MDD patients with sarcopenia comorbidity would present higher suicide risk than those without. Since high suicide rates are a popular problem in the non-elderly, and muscle health is age-related, we only studied non-elderly adults with MDD. In order to minimize the effect of antidepressants and recurrence, we only included first-episode drug-naïve MDD patients. This study aimed to explore if sarcopenia was associated with severity of suicide risk in non-elderly inpatients with MDD after adjustment for depression severity.

## Methods

### Study population

This was a cross-sectional study targeting first-episode drug-naïve MDD inpatients at the psychiatric department of the Second Affiliated Hospital of Kunming Medical University from January to December 2018. We included patients aged 20–59 years who met the criteria of MDD based on the Diagnostic and Statistical Manual of Mental Disorders, Fourth Edition (DSM-IV) with the total scores of the 24-item Hamilton rating scale for depression (HAMD-24) greater than 20 points. The exclusion criteria were current hyperthyroidism, hypothyroidism, mental retardation, chronic or acute heart or renal failure, hepatic insufficiency, pregnancy, cancer, chronic serious physical disabilities, implanted electrical devices, home oxygen therapy, taking long-term systemic corticosteroids and inability to complete self-administered tests or self-rating scales independently. Demographic information and medical history were collected, including gender, age and years of education. All the doctor-administered clinical assessments were completed by two trained psychiatrists.

### Assessment of depression related scales

The HAMD-24 was used to assess the severity of depression and all the participants in this study scored greater than 20 points [22]. The Hamilton Rating Scale for Anxiety (HAMA) was used to assess the severity of anxiety [23]. The Nurses’ Global Assessment of Suicide Risk (NGASR) was used to assess the suicide risk of the participants [24]. It is a risk assessment tool of suicide, incorporating key indicators of suicide based on current evidence regarding suicide. There are 15 items: feelings of hopelessness, recent stressful life events, persecutory voices/beliefs, depression/loss of interest, withdrawal from interpersonal and social interactions, verbalization of suicide intent, a plan to commit suicide, family history of severe psychiatric problems or suicide, recent bereavement or relationship breakdown, personal history of psychosis, widow/widower, prior suicide attempt, history of socio-economic deprivation, history of alcohol and/or alcohol misuse, and presence of a terminal illness. The suicide risk in this manuscript determined by NGASR means the risk of committing suicide. A total score of ≤5, 6–8, 9–11 and ≥ 12 points were classified as low, intermediate, high and very high suicide risk, respectively. The Chinese version of NGASR was proved to have good reliability and content validity according to Beck Hopelessness Scale (BSS) and Beck Scale for Suicide Ideation (BSI) [25, 26]. Currently NGASR is widely used in clinical practice and psychiatric researches in inpatients of psychiatric departments [27–30].

### Laboratory assays

Blood samples were collected from the antecubital vein after overnight fasting. Plasma cortisol and adrenocorticotropic hormone (ACTH) levels were measured by chemiluminescence immunoassay (AutoLumo A2000).

### Assessment of sarcopenia

Sarcopenia was evaluated by the SARC-F questionnaire. It is a 5-item mnemonic with each capital letter presenting an item for assessing – Strength, Assistance in walking, Rise from a chair, Climb stairs and Falls. Each item is scored ranging from 0 to 2 points. In 2013, Malmstrom and Morley developed this questionnaire to screen sarcopenia simply, rapidly and conveniently, and suggested that a total score of ≥4 points was considered to indicate sarcopenia [31]. The SARC-F has been validated in different ethnic populations and countries including Chinese, and has been proven valid and consistent for identifying those at risk of sarcopenia in these populations [13], with high specificity but relatively low sensitivity [32–35].

### Statistical analyses

Statistical analyses were performed by SPSS 25.0 (Statistical Package for Social Sciences, SPSS inc). Continuous data were presented as mean ± standard deviation (SD) if normally distributed, or as median [interquartile range] if skewedly distributed. Categorical data were presented as absolute numbers and percentages. Demographic and clinical characteristics were compared across different severities of suicide risk with the categorical data by *χ*^2^ tests or Fisher’s exact tests and continuous data by one-way ANOVA or Kruskal-Wallis H rank sum tests. Ordinal regression models were then used to evaluate the factors associated with severity of suicide risk. Statistical significance was defined as a two-sided *p* value of less than 0.05.

## Results

Of the 245 MDD inpatients who met the inclusion criteria, 25 were excluded and 28 had missing data. The remaining 192 first-episode drug-naïve MDD inpatients (122 female and 70 males; aged 39.3 ± 11.7 years) were analyzed in this study.

Among the 192 inpatients included in this study, 69 (35.9%) were grouped in the low risk group, 76 (39.6%) in the intermedium risk group, 21 (10.9%) in the high risk group and 26 (13.5%) in the very high risk group. 24 (12.5%) inpatients were screened as having sarcopenia, including 4 males (5.7%) and 20 females (16.4%), with significant difference in gender (*p* = 0.040). Table 1 summarizes the comparisons of demographic and clinical characteristics by severity of suicide risk. There were significant differences in gender, HAMD score and prevalence of sarcopenia among the suicide risk groups, while no significant differences were found in age, years of education, disease duration, HAMA score, cortisol level and ACTH level.

**Table 1:** Comparison of the characteristics by severity of suicide risk

Variables	Low *N* = 69	Intermedium *N* = 76	High *N* = 21	Very high *N* = 26	*P*
Age (years)	40.7 ± 11.4	40.1 ± 11.2	39.0 ± 13.0	33.9 ± 11.9	0.075
Gender (Female)	39 (56.5%)	45 (59.2%)	15 (71.4%)	23 (63.5%)	0.004*
Education (years)	12.8 ± 4.2	12.8 ± 3.9	11.5 ± 5.1	13.2 ± 3.1	0.526
Cortisol (μg/dL)	19.33 ± 8.01	21.46 ± 10.52	21.88 ± 8.41	21.08 ± 8.57	0.487
ACTH (pg/mL)	32.54 [36.87]	32.79 [32.65]	39.23 [34.11]	32.05 [30.86]	0.520
HAMD score	26.0 ± 4.7	30.3 ± 7.9	36.8 ± 6.2	38.9 ± 6.1	<0.001**
HAMA score	16.6 ± 7.5	17.0 ± 8.7	20.5 ± 8.3	19.2 ± 9.7	0.187
Sarcopenia	2 (2.9%)	10 (13.2%)	6 (28.6%)	6 (23.1%)	0.001*

Data are presented as mean ± SD, median [interquartile range] or absolute numbers (percentage)

* *p* < 0.05; ** *p* < 0.001

The results of unadjusted and adjusted ordinal regression models using severities of suicide risk as the dependent variable and other variables as the explanatory variables were shown in Table 2. In the unadjusted model, sarcopenia was significantly associated with severer suicide risk (OR = 3.85, 95%CI 1.73–8.56, *p* = 0.001). After adjustment for HAMD score, age and gender, sarcopenia remained significantly associated with severer suicide risk (OR = 2.39, 95%CI 1.02–5.58, *p* = 0.044).

**Table 2:** Association of suicide risk with sarcopenia in unadjusted and adjusted ordinal regression models

Variables	Unadjusted OR (95% CI)	*P*	Model 1 OR (95% CI)	*P*	Model 2 OR (95% CI)	*P*
Sarcopenia	3.85 (1.73–8.56)	0.001*	2.36 (1.01–5.50)	0.047*	2.39 (1.02–5.58)	0.044*
HAMD score			1.16 (1.12–1.21)	<0.001**	1.16 (1.11–1.21)	<0.001**
Male			0.74 (0.41–1.33)	0.311	0.73 (0.40–1.31)	0.288
Age					0.99 (0.97–1.02)	0.621

Model 1: adjusted for HAMD score and gender

Model 2: adjusted for HAMD score, gender and age

OR = odds of being in a more severe suicide risk group

* *p* < 0.05; ** *p* < 0.001

## Discussion

The aim of the study was to investigate whether sarcopenia was associated with severity of suicide risk in non-elderly inpatients with MDD after adjustment for depression severity. The results suggest that those with severer depression and sarcopenia comorbidity have higher suicide risk, and sarcopenia is significantly associated with severity of suicide risk even in the significant context of depression severity, which indicate that sarcopenia screening and identification is essential in MDD patients.

Our study also showed that in non-elderly MDD patients, females tend to have higher suicide risk than males, consistent with some previous studies [36]. While another study found males did more suicides than females [37], which may attribute to the difference in suicide assessments. As to the relationship between HPA axis and suicide risk, it has been suggested that stressful psychological events may be used to predict suicide risk [38], and the function of HPA axis has been reported to be associated with suicide through regulating the response to stress, but the results are inconsistent [39–42]. Our study found no significant differences in ACTH and cortisol levels across suicide risk severity groups. So prospective studies with large sample and consistent study design may be needed to explore the relationship.

Previous studies have reported associations between suicide and muscle-related indicators. Specifically, suicide was found positively associated with decreased skeletal muscle strength [8–10, 43], poorer physical performance [9], sarcopenia [44, 45] and physical inactivity [46, 47]. In line with these researches, our study found that sarcopenia, a skeletal muscle disorder, was associated with higher suicide risk. Therefore, mechanisms of the relationship between sarcopenia and suicide deserve further investigation.

Current researches suggest some underlying mechanisms for the association between suicide and muscle. From the perspective of psychosocial factors, decreased muscle strength was associated with lowered self-concept and self-esteem [48, 49] that could increase suicide risk, while physical exercise may both benefit those with sarcopenia and those with high suicide risk [46, 50], from which we could infer that sarcopenia is associated with suicide, consistent with our result.

The association might also be explained from the perspective of biological factors. A study on biomarkers of suicide by Niculescu et al. have shown that patients with higher suicide risk have relatively high levels of interleukin-6 (IL-6) [21] that involved in inflammation response. While chronic low-grade inflammation might increase muscle catabolism, which is one of the pathophysiological characteristics of sarcopenia [51]. Further, these pro-inflammatory cytokines can activate kynurenine pathway [52–54], increase the level of quinolinic acid, the NMDA receptor agonist, and simultaneously decrease neuroprotective metabolites via glutamate neurotransmission and neuroinflammation [55]. Whereas physical exercise induces the release of PGC-1α1 in skeletal muscle that increases the expression of kynurenine aminotransferases, thus promoting the conversion of kynurenine into kynurenic acid, a metabolite unable to pass the blood-brain barrier, and protecting the brain from stress-induced changes, which can be instrumental in relieving depression and suicide ideation [56]. Moreover, BDNF, a key regulator of brain development and plasticity, is related to suicide risk when it is dysfunctional [19]. While physical exercise mediates central BDNF production in humans [57], and have positive effects on brain plasticity and increases brain reserve capacity, which might reduce suicide risk [58, 59]. In brief, the relationship between sarcopenia and suicide may be linked by inflammation, kynurenine metabolism and brain plasticity, and potential intervention in these factors might not only relieve depression and suicide ideation, but also improve sarcopenia, as our results suggest.

### Strengths and limitations

To the best of our knowledge, this was the first study to reveal that sarcopenia, screened by the recently developed simple SARC-F questionnaire, was associated with suicide risk severity in non-elderly fist-episode drug-naïve MDD inpatients. Early identifying and intervention for sarcopenia might ameliorate suicide risk in MDD patients.

There existed a number of limitations in this study. First, the SARC-F questionnaire was used as the way of identifying sarcopenia in this study. However, SARC-F was a simple screening tool rather than a diagnostic criterion for sarcopenia, with relatively high specificity and low-to-moderate sensitivity, which might affect the validity of this study. Accordingly, those identified as sarcopenia should have further test for body composition in order to make a definite diagnosis. Second, more females and the small sample size of sarcopenia in this study might lead to biased results and limit the generality and explanatory power. Additionally, as this was a cross-sectional study, the causal relationship between sarcopenia and suicide risk was unable to determine. Whether sarcopenia could affect the efficacy of antidepressant therapy or worsen the prognosis is worth further studying. Therefore, future prospective studies with larger samples should be conducted among MDD patients with comorbid sarcopenia at different ages to explore whether there will be therapeutic response and brain changes together with or dependent of depression treatment after intervention of sarcopenia.

## Conclusions

This study revealed that sarcopenia was significantly associated with higher suicide risk in non-elderly MDD inpatients after adjustment for depression severity. Educating psychiatric staffs about sarcopenia screening, identifying and intervening is important. Improving the patients’ physical fitness might help MDD patients to reduce suicide risk and get better social rehabilitation. Future prospective researches are needed to elucidate whether suicide risk could be reduced through intervention of sarcopenia.

## Data Availability

The data used in the current study are available from the corresponding author for reasonable request.

## Abbreviations

ACTH: Adrenocorticotropic hormone
BDNF: Brain-derived neurotropic factor
BSI: Beck Scale for Suicide Ideation
BSS: Beck Hopelessness Scale
DSM-IV: Diagnostic and Statistical Manual of Mental Disorders, Fourth Edition
HAMD-24: 24-item Hamilton rating scale for depression
HAMA: Hamilton Rating Scale for Anxiety
HPA: Hypothalamic-pituitary-adrenal
IL-6: Interleukin-6
MDD: Major depressive disorder
NGASR: 15-item Nurses’ Global Assessment of Suicide Risk
SD: Standard deviation
